# 1-Methyl-7-(4-nitro­phen­yl)-3-phenyl­pyrazolo[3,4-*b*]pyrrolo[3,4-*d*]pyridine-6,8(3*H*,7*H*)-dione

**DOI:** 10.1107/S1600536808037240

**Published:** 2008-11-13

**Authors:** Jose R. Sabino, Cecília M. A. Oliveira, Carlos A. M. Fraga, Eliezer J. Barreiro, Valéria de Oliveira, Ricardo Menegatti

**Affiliations:** a Instituto de Física, Universidade Federal de Goiás, Caixa Postal 131, 74001-970, Goiânia, GO, Brazil; b Instituto de Química, Universidade Federal de Goiás, Caixa Postal 131, 74001-970, Goiânia, GO, Brazil; c Laboratory of Evaluation and Synthesis of Bioactive Substances, (LASSBio), Faculdade de Farmácia, Universidade Federal do Rio de Janeiro, Caixa Postal 68023, 21944-971, Rio de Janeiro, RJ, Brazil; d Faculdade de Farmácia, Universidade Federal de Goiás, Caixa Postal 131, 74001-970, Goiânia, GO, Brazil

## Abstract

In the title compound, C_21_H_13_N_5_O_4_, the dihedral angles formed between the planes of the phenyl and nitro­phenyl rings and that of the heterotricyclic plane are 41.29 (7) and 61.35 (6)°, respectively. In the crystal, weak C—H⋯O interactions help to establish the packing.

## Related literature

For background, see: Carneiro *et al.* (2005 ▶); Menegatti *et al.* (2006 ▶); Barreiro *et al.* (2006 ▶).
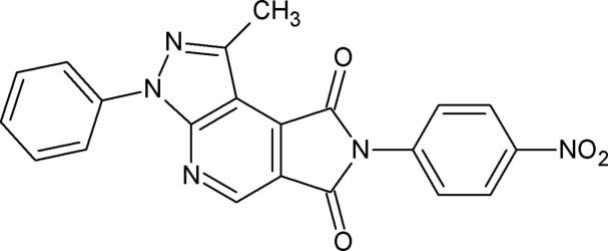


## Experimental

### 

#### Crystal data

C_21_H_13_N_5_O_4_
*M* *_r_* = 399.36
Monoclinic, 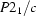

*a* = 9.677 (2) Å
*b* = 12.141 (3) Å
*c* = 17.438 (4) Å
β = 119.451 (16)°
*V* = 1784.0 (8) Å^3^
*Z* = 4
Cu *K*α radiation
μ = 0.89 mm^−1^
*T* = 297 (2) K
0.3 × 0.1 × 0.08 mm

#### Data collection

Enraf–Nonius CAD-4 diffractometer
Absorption correction: none
3813 measured reflections
3259 independent reflections
2578 reflections with *I* > 2σ(*I*)
*R*_int_ = 0.023
2 standard reflections frequency: 120 min intensity decay: 1%

#### Refinement

*R*[*F* ^2^ > 2σ(*F* ^2^)] = 0.049
*wR*(*F* ^2^) = 0.198
*S* = 1.07
3259 reflections
273 parameters
H-atom parameters constrained
Δρ_max_ = 0.48 e Å^−3^
Δρ_min_ = −0.38 e Å^−3^

### 

Data collection: *CAD-4-PC* (Enraf–Nonius, 1993 ▶); cell refinement: *CAD-4-PC*; data reduction: *XCAD4* (Harms & Wocadlo, 1995 ▶); program(s) used to solve structure: *SHELXS97* (Sheldrick, 2008 ▶); program(s) used to refine structure: *SHELXL97* (Sheldrick, 2008 ▶); molecular graphics: *PLATON* (Spek, 2003 ▶); software used to prepare material for publication: *WinGX* (Farrugia, 1999 ▶).

## Supplementary Material

Crystal structure: contains datablocks global, I. DOI: 10.1107/S1600536808037240/tk2318sup1.cif

Structure factors: contains datablocks I. DOI: 10.1107/S1600536808037240/tk2318Isup2.hkl

Additional supplementary materials:  crystallographic information; 3D view; checkCIF report

## Figures and Tables

**Table 1:** Hydrogen-bond geometry (Å, °)

*D*—H⋯*A*	*D*—H	H⋯*A*	*D*⋯*A*	*D*—H⋯*A*
C7—H7⋯O30^i^	0.93	2.55	3.216 (3)	129
C18—H18⋯O30^ii^	0.93	2.44	3.197 (3)	139

Symmetry codes: (i) 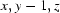; (ii) 
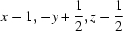
.

**Fig. 1.**
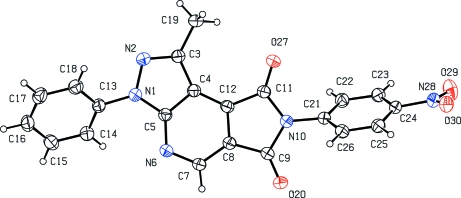
View of (I) with the atom-numbering scheme. Displacement ellipsoids are drawn at the 30% probability level and H atoms are shown as small spheres of arbitrary radii.

**Fig. 2.**
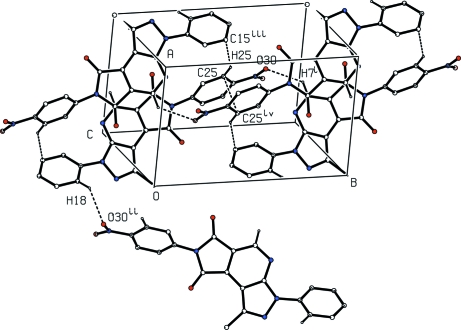
Packing diagram of (I). Intermolecular interactions are shown as dashed lines. Only the H atoms involved in intermolecular contacts are shown.

## supplementary crystallographic information

### Comment

The title compound (I) (Carneiro *et al.*, 2005; Menegatti *et al.*, 2006; Barreiro *et al.*, 2006) features a novel functionalized three-fused rings skeleton, Fig. 1. The bond distances within the pyridine ring are consistent with aromatic delocalization but the pyrazole ring presents a double bond, Table 1. The C8—C9 and C11—C12 bond lengths are elongated from the expected distances by 0.025 and 0.03 Å, respectively.

The pyrazolo[3,4-*b*]pyrrolo[3,4-*d*]pyridine moiety is planar (plane α, with r.m.s. deviation of about 0.01 Å). Relative to plane α, the phenyl rings C21/C26 and C13/C18 form dihedral angles of 41.29 (7)° and 61.35 (6)°, respectively. The latter ring is slanted from the plane α so that the C21 and C24 atoms are shifted by 0.118 (3) and 0.436 (4) Å from the plane α, respectively. This might be in part due to the participation of the oxygen atom O30 in two C-H···O contacts, C7—H7···O30^i^ and C18—H18···O30^ii^; see Table 2 for details. The former interaction connects molecules into a linear chain along the b-axis and the latter connects parallel chains to form sheets in the plane of approximate indices (-1 0 2). The molecules in the linear chains interact with centrosymmetrically related chains via π···π stacking [closest distance for C25···C25^iv^: 3.380 (5) Å; (iv): 1 - *x*, 1 - *y*, 1 - *z*]. In addition, molecules participate in a C—H···π interaction: C25—H25···C15^iii ^ [2.74 Å, 3.539 (4) Å, 144°; (iii) 1 -*x*, -*y*, 1 -*z*] to consolidate the crystal packing, Fig. 2.

### Experimental

Compound (I) was synthesized as described previously (Menegatti *et al.*, 2006). A colourless needle for crystallography was obtained by slow evaporation of a MeOH solution of (I) at room temperature.

### Refinement

All H atoms were positioned in idealized positions in the riding model approximation with C—H = 0.93 - 0.96 Å, and with *U*_iso_(H) = 1.2-1.5 *U*_eq_(C).

### Crystal data

**Table:**

C_21_H_13_N_5_O_4_	*F*_000_ = 824
*M**_r_* = 399.36	*D*_x_ = 1.487 Mg m^−^^3^
Monoclinic, *P*2_1_/*c*	Cu *K*α radiation λ = 1.5418 Å
Hall symbol: -P 2ybc	Cell parameters from 25 reflections
*a* = 9.677 (2) Å	θ = 14.8–40.8º
*b* = 12.141 (3) Å	µ = 0.89 mm^−^^1^
*c* = 17.438 (4) Å	*T* = 297 (2) K
β = 119.451 (16)º	Prism, colourless
*V* = 1784.0 (8) Å^3^	0.3 × 0.1 × 0.08 mm
*Z* = 4	

### Data collection

**Table:**

Enraf–Nonius CAD-4 diffractometer	θ_max_ = 67.9º
*T* = 297(2) K	θ_min_ = 4.7º
Non–profiled ω/2ω scans	*h* = 0→11
Absorption correction: none	*k* = −14→1
3813 measured reflections	*l* = −20→18
3259 independent reflections	2 standard reflections
2578 reflections with *I* > 2σ(*I*)	every 120 min
*R*_int_ = 0.023	intensity decay: 1%

### Refinement

**Table:**

Refinement on *F*^2^	H-atom parameters constrained
Least-squares matrix: full	*w* = 1/[σ^2^(*F*_o_^2^) + (0.152*P*)^2^ + 0.0892*P*] where *P* = (*F*_o_^2^ + 2*F*_c_^2^)/3
*R*[*F*^2^ > 2σ(*F*^2^)] = 0.049	(Δ/σ)_max_ < 0.001
*wR*(*F*^2^) = 0.198	Δρ_max_ = 0.48 e Å^−^^3^
*S* = 1.07	Δρ_min_ = −0.38 e Å^−^^3^
3259 reflections	Extinction correction: SHELXL97 (Sheldrick, 2008), Fc^*^=kFc[1+0.001xFc^2^λ^3^/sin(2θ)]^-1/4^
273 parameters	Extinction coefficient: 0.0114 (15)

### Special details

**Table:**

Geometry. All e.s.d.'s (except the e.s.d. in the dihedral angle between two l.s. planes) are estimated using the full covariance matrix. The cell e.s.d.'s are taken into account individually in the estimation of e.s.d.'s in distances, angles and torsion angles; correlations between e.s.d.'s in cell parameters are only used when they are defined by crystal symmetry. An approximate (isotropic) treatment of cell e.s.d.'s is used for estimating e.s.d.'s involving l.s. planes.

### Fractional atomic coordinates and isotropic or equivalent isotropic displacement parameters (Å^2^)

**Table:**

	*x*	*y*	*z*	*U*_iso_*/*U*_eq_	
C3	−0.0408 (3)	0.00360 (19)	0.37884 (14)	0.0446 (5)	
C4	0.1214 (3)	0.00403 (18)	0.44674 (14)	0.0425 (5)	
C5	0.1708 (3)	−0.10707 (19)	0.45566 (14)	0.0431 (5)	
C7	0.4230 (3)	−0.07508 (19)	0.56234 (16)	0.0500 (6)	
H7	0.5254	−0.0987	0.6016	0.06*	
C8	0.3874 (3)	0.03680 (18)	0.55955 (14)	0.0452 (5)	
C9	0.4917 (3)	0.13014 (18)	0.61003 (15)	0.0455 (5)	
C11	0.2390 (3)	0.19832 (18)	0.51522 (14)	0.0442 (5)	
C12	0.2381 (3)	0.07694 (18)	0.50241 (14)	0.0427 (5)	
C13	0.0263 (3)	−0.28159 (19)	0.37880 (16)	0.0462 (5)	
C14	0.0917 (3)	−0.3569 (2)	0.44682 (16)	0.0514 (6)	
H14	0.1508	−0.3332	0.5049	0.062*	
C15	0.0681 (3)	−0.4684 (2)	0.42733 (19)	0.0584 (7)	
H15	0.1132	−0.5198	0.4726	0.07*	
C16	−0.0219 (3)	−0.5039 (2)	0.3414 (2)	0.0606 (7)	
H16	−0.0394	−0.5787	0.3289	0.073*	
C17	−0.0852 (3)	−0.4279 (2)	0.27450 (19)	0.0616 (7)	
H17	−0.1454	−0.4516	0.2165	0.074*	
C18	−0.0604 (3)	−0.3157 (2)	0.29235 (16)	0.0542 (6)	
H18	−0.1016	−0.2645	0.2468	0.065*	
C19	−0.1519 (3)	0.0984 (2)	0.34157 (17)	0.0549 (6)	
H19A	−0.2592	0.0727	0.3172	0.082*	
H19B	−0.1383	0.1329	0.2962	0.082*	
H19C	−0.13	0.1509	0.3874	0.082*	
C21	0.4456 (3)	0.33472 (18)	0.60655 (14)	0.0426 (5)	
C22	0.3722 (3)	0.3988 (2)	0.64181 (16)	0.0489 (5)	
H22	0.2948	0.3685	0.6523	0.059*	
C23	0.4150 (3)	0.5083 (2)	0.66135 (16)	0.0507 (6)	
H23	0.3682	0.5525	0.6859	0.061*	
C24	0.5284 (3)	0.55016 (18)	0.64357 (14)	0.0461 (5)	
C25	0.6055 (3)	0.4867 (2)	0.61069 (15)	0.0496 (6)	
H25	0.6842	0.5169	0.6012	0.06*	
C26	0.5631 (3)	0.37726 (19)	0.59215 (15)	0.0480 (5)	
H26	0.6134	0.3325	0.5701	0.058*	
O20	0.6301 (2)	0.12980 (14)	0.66441 (12)	0.0587 (5)	
O27	0.1327 (2)	0.26369 (14)	0.47771 (13)	0.0592 (5)	
O30	0.6424 (2)	0.70822 (16)	0.62526 (14)	0.0675 (6)	
N1	0.0428 (2)	−0.16562 (16)	0.39668 (12)	0.0469 (5)	
N2	−0.0858 (2)	−0.09733 (17)	0.34974 (13)	0.0497 (5)	
N6	0.3155 (2)	−0.14946 (16)	0.51075 (13)	0.0489 (5)	
N10	0.3932 (2)	0.22413 (15)	0.58107 (12)	0.0453 (5)	
N28	0.5686 (3)	0.66741 (18)	0.65876 (13)	0.0561 (6)	
O29	0.5258 (3)	0.71978 (17)	0.70287 (16)	0.0830 (7)	

### Atomic displacement parameters (Å^2^)

**Table:**

	*U*^11^	*U*^22^	*U*^33^	*U*^12^	*U*^13^	*U*^23^
C3	0.0414 (11)	0.0460 (11)	0.0430 (11)	0.0022 (9)	0.0181 (9)	0.0009 (9)
C4	0.0394 (11)	0.0436 (11)	0.0430 (11)	0.0017 (9)	0.0190 (9)	0.0022 (9)
C5	0.0401 (11)	0.0427 (11)	0.0459 (11)	0.0016 (9)	0.0207 (9)	0.0017 (9)
C7	0.0398 (11)	0.0408 (11)	0.0580 (13)	0.0049 (9)	0.0152 (10)	0.0057 (9)
C8	0.0418 (11)	0.0406 (11)	0.0479 (11)	0.0017 (9)	0.0180 (10)	0.0039 (9)
C9	0.0436 (12)	0.0393 (12)	0.0490 (12)	0.0048 (9)	0.0192 (10)	0.0033 (9)
C11	0.0407 (11)	0.0425 (12)	0.0464 (11)	0.0035 (9)	0.0191 (9)	0.0036 (9)
C12	0.0410 (11)	0.0411 (11)	0.0444 (11)	0.0046 (9)	0.0196 (9)	0.0045 (8)
C13	0.0411 (11)	0.0424 (12)	0.0567 (12)	−0.0020 (9)	0.0252 (10)	−0.0056 (9)
C14	0.0511 (13)	0.0476 (13)	0.0544 (13)	−0.0011 (10)	0.0250 (11)	−0.0023 (10)
C15	0.0586 (15)	0.0462 (13)	0.0755 (17)	0.0040 (11)	0.0368 (13)	0.0043 (12)
C16	0.0550 (14)	0.0457 (13)	0.0872 (19)	−0.0057 (11)	0.0397 (14)	−0.0145 (12)
C17	0.0524 (14)	0.0661 (17)	0.0650 (15)	−0.0068 (12)	0.0280 (12)	−0.0226 (13)
C18	0.0492 (13)	0.0567 (14)	0.0548 (13)	−0.0003 (11)	0.0241 (11)	−0.0034 (11)
C19	0.0426 (12)	0.0534 (14)	0.0545 (13)	0.0090 (10)	0.0130 (11)	0.0022 (10)
C21	0.0389 (11)	0.0386 (11)	0.0436 (10)	0.0011 (8)	0.0152 (9)	0.0014 (8)
C22	0.0469 (12)	0.0464 (12)	0.0550 (12)	−0.0016 (10)	0.0264 (11)	−0.0028 (10)
C23	0.0475 (12)	0.0483 (12)	0.0530 (12)	0.0023 (10)	0.0222 (10)	−0.0079 (10)
C24	0.0420 (11)	0.0390 (11)	0.0441 (11)	−0.0020 (9)	0.0109 (9)	−0.0046 (9)
C25	0.0441 (12)	0.0463 (12)	0.0581 (13)	−0.0018 (9)	0.0248 (11)	0.0008 (10)
C26	0.0454 (12)	0.0457 (12)	0.0528 (12)	0.0029 (10)	0.0241 (10)	−0.0026 (10)
O20	0.0414 (9)	0.0510 (10)	0.0623 (11)	0.0035 (7)	0.0089 (8)	0.0036 (7)
O27	0.0457 (9)	0.0434 (9)	0.0709 (11)	0.0100 (7)	0.0152 (8)	0.0002 (8)
O30	0.0607 (11)	0.0490 (10)	0.0811 (13)	−0.0093 (9)	0.0258 (10)	0.0001 (9)
N1	0.0415 (10)	0.0421 (10)	0.0497 (10)	−0.0009 (8)	0.0167 (8)	−0.0029 (8)
N2	0.0411 (10)	0.0498 (11)	0.0498 (10)	0.0019 (8)	0.0159 (9)	−0.0013 (8)
N6	0.0420 (10)	0.0395 (9)	0.0568 (11)	0.0039 (8)	0.0178 (9)	0.0014 (8)
N10	0.0408 (10)	0.0379 (10)	0.0493 (10)	0.0013 (8)	0.0161 (8)	0.0012 (7)
N28	0.0528 (12)	0.0460 (11)	0.0535 (11)	−0.0040 (9)	0.0139 (10)	−0.0058 (9)
O29	0.1122 (19)	0.0534 (11)	0.0905 (15)	−0.0043 (12)	0.0553 (15)	−0.0207 (10)

### Geometric parameters (Å, °)

**Table:**

C3—N2	1.316 (3)	C16—C17	1.373 (4)
C3—C4	1.428 (3)	C16—H16	0.93
C3—C19	1.489 (3)	C17—C18	1.392 (4)
C4—C12	1.387 (3)	C17—H17	0.93
C4—C5	1.414 (3)	C18—H18	0.93
C5—N6	1.351 (3)	C19—H19A	0.96
C5—N1	1.358 (3)	C19—H19B	0.96
C7—N6	1.337 (3)	C19—H19C	0.96
C7—C8	1.396 (3)	C21—C26	1.380 (3)
C7—H7	0.93	C21—C22	1.385 (3)
C8—C12	1.379 (3)	C21—N10	1.427 (3)
C8—C9	1.484 (3)	C22—C23	1.384 (3)
C9—O20	1.202 (3)	C22—H22	0.93
C9—N10	1.411 (3)	C23—C24	1.376 (4)
C11—O27	1.206 (3)	C23—H23	0.93
C11—N10	1.400 (3)	C24—C25	1.378 (3)
C11—C12	1.490 (3)	C24—N28	1.465 (3)
C13—C18	1.380 (3)	C25—C26	1.382 (3)
C13—C14	1.380 (3)	C25—H25	0.93
C13—N1	1.434 (3)	C26—H26	0.93
C14—C15	1.387 (3)	O30—N28	1.228 (3)
C14—H14	0.93	N1—N2	1.380 (3)
C15—C16	1.381 (4)	N28—O29	1.216 (3)
C15—H15	0.93		
			
N2—C3—C4	110.1 (2)	C13—C18—C17	118.9 (2)
N2—C3—C19	121.4 (2)	C13—C18—H18	120.6
C4—C3—C19	128.5 (2)	C17—C18—H18	120.6
C12—C4—C5	114.68 (19)	C3—C19—H19A	109.5
C12—C4—C3	140.1 (2)	C3—C19—H19B	109.5
C5—C4—C3	105.19 (19)	H19A—C19—H19B	109.5
N6—C5—N1	125.4 (2)	C3—C19—H19C	109.5
N6—C5—C4	128.1 (2)	H19A—C19—H19C	109.5
N1—C5—C4	106.44 (19)	H19B—C19—H19C	109.5
N6—C7—C8	122.5 (2)	C26—C21—C22	121.3 (2)
N6—C7—H7	118.8	C26—C21—N10	119.6 (2)
C8—C7—H7	118.8	C22—C21—N10	119.1 (2)
C12—C8—C7	121.5 (2)	C23—C22—C21	119.5 (2)
C12—C8—C9	108.9 (2)	C23—C22—H22	120.2
C7—C8—C9	129.6 (2)	C21—C22—H22	120.2
O20—C9—N10	125.4 (2)	C24—C23—C22	118.3 (2)
O20—C9—C8	129.4 (2)	C24—C23—H23	120.8
N10—C9—C8	105.23 (18)	C22—C23—H23	120.8
O27—C11—N10	125.4 (2)	C23—C24—C25	122.8 (2)
O27—C11—C12	129.0 (2)	C23—C24—N28	119.2 (2)
N10—C11—C12	105.65 (18)	C25—C24—N28	117.9 (2)
C8—C12—C4	118.9 (2)	C24—C25—C26	118.4 (2)
C8—C12—C11	108.3 (2)	C24—C25—H25	120.8
C4—C12—C11	132.8 (2)	C26—C25—H25	120.8
C18—C13—C14	121.1 (2)	C21—C26—C25	119.6 (2)
C18—C13—N1	118.3 (2)	C21—C26—H26	120.2
C14—C13—N1	120.6 (2)	C25—C26—H26	120.2
C13—C14—C15	119.0 (2)	C5—N1—N2	110.82 (18)
C13—C14—H14	120.5	C5—N1—C13	129.86 (19)
C15—C14—H14	120.5	N2—N1—C13	119.32 (18)
C16—C15—C14	120.7 (2)	C3—N2—N1	107.44 (18)
C16—C15—H15	119.6	C7—N6—C5	114.4 (2)
C14—C15—H15	119.6	C11—N10—C9	111.87 (18)
C17—C16—C15	119.4 (2)	C11—N10—C21	122.56 (18)
C17—C16—H16	120.3	C9—N10—C21	125.15 (19)
C15—C16—H16	120.3	O29—N28—O30	123.2 (2)
C16—C17—C18	120.8 (3)	O29—N28—C24	118.7 (2)
C16—C17—H17	119.6	O30—N28—C24	118.1 (2)
C18—C17—H17	119.6		
			
N2—C3—C4—C12	179.4 (3)	C22—C23—C24—N28	176.4 (2)
C19—C3—C4—C12	−0.2 (5)	C23—C24—C25—C26	2.2 (4)
N2—C3—C4—C5	0.3 (3)	N28—C24—C25—C26	−177.0 (2)
C19—C3—C4—C5	−179.4 (2)	C22—C21—C26—C25	−2.0 (3)
C12—C4—C5—N6	−0.3 (3)	N10—C21—C26—C25	175.2 (2)
C3—C4—C5—N6	179.1 (2)	C24—C25—C26—C21	0.2 (3)
C12—C4—C5—N1	−179.93 (19)	N6—C5—N1—N2	−179.1 (2)
C3—C4—C5—N1	−0.5 (2)	C4—C5—N1—N2	0.6 (2)
N6—C7—C8—C12	−0.4 (4)	N6—C5—N1—C13	1.1 (4)
N6—C7—C8—C9	−178.5 (2)	C4—C5—N1—C13	−179.2 (2)
C12—C8—C9—O20	−178.1 (3)	C18—C13—N1—C5	−140.1 (2)
C7—C8—C9—O20	0.2 (4)	C14—C13—N1—C5	41.9 (4)
C12—C8—C9—N10	2.0 (3)	C18—C13—N1—N2	40.1 (3)
C7—C8—C9—N10	−179.7 (2)	C14—C13—N1—N2	−137.9 (2)
C7—C8—C12—C4	0.0 (3)	C4—C3—N2—N1	0.1 (3)
C9—C8—C12—C4	178.5 (2)	C19—C3—N2—N1	179.8 (2)
C7—C8—C12—C11	−179.7 (2)	C5—N1—N2—C3	−0.4 (3)
C9—C8—C12—C11	−1.2 (2)	C13—N1—N2—C3	179.4 (2)
C5—C4—C12—C8	0.3 (3)	C8—C7—N6—C5	0.4 (4)
C3—C4—C12—C8	−178.8 (3)	N1—C5—N6—C7	179.5 (2)
C5—C4—C12—C11	179.9 (2)	C4—C5—N6—C7	−0.1 (4)
C3—C4—C12—C11	0.9 (5)	O27—C11—N10—C9	−178.1 (2)
O27—C11—C12—C8	179.4 (2)	C12—C11—N10—C9	1.3 (2)
N10—C11—C12—C8	0.0 (2)	O27—C11—N10—C21	−5.2 (4)
O27—C11—C12—C4	−0.3 (4)	C12—C11—N10—C21	174.2 (2)
N10—C11—C12—C4	−179.6 (2)	O20—C9—N10—C11	178.1 (2)
C18—C13—C14—C15	−0.5 (4)	C8—C9—N10—C11	−2.0 (3)
N1—C13—C14—C15	177.4 (2)	O20—C9—N10—C21	5.4 (4)
C13—C14—C15—C16	−1.1 (4)	C8—C9—N10—C21	−174.7 (2)
C14—C15—C16—C17	1.5 (4)	C26—C21—N10—C11	−115.1 (2)
C15—C16—C17—C18	−0.3 (4)	C22—C21—N10—C11	62.1 (3)
C14—C13—C18—C17	1.8 (4)	C26—C21—N10—C9	56.9 (3)
N1—C13—C18—C17	−176.2 (2)	C22—C21—N10—C9	−125.9 (2)
C16—C17—C18—C13	−1.4 (4)	C23—C24—N28—O29	14.8 (3)
C26—C21—C22—C23	1.4 (4)	C25—C24—N28—O29	−166.1 (2)
N10—C21—C22—C23	−175.8 (2)	C23—C24—N28—O30	−164.4 (2)
C21—C22—C23—C24	1.0 (4)	C25—C24—N28—O30	14.8 (3)
C22—C23—C24—C25	−2.8 (4)		

### Hydrogen-bond geometry (Å, °)

**Table:**

*D*—H···*A*	*D*—H	H···*A*	*D*···*A*	*D*—H···*A*
C7—H7···O30^i^	0.93	2.55	3.216 (3)	129
C18—H18···O30^ii^	0.93	2.44	3.197 (3)	139

Symmetry codes: (i) *x*, *y*−1, *z*; (ii) *x*−1, −*y*+1/2, *z*−1/2.
